# Selection of Novel Cowpea Genotypes Derived through Gamma Irradiation

**DOI:** 10.3389/fpls.2016.00262

**Published:** 2016-03-10

**Authors:** Lydia N. Horn, Habteab M. Ghebrehiwot, Hussein A. Shimelis

**Affiliations:** ^1^School of Agricultural, Earth and Environmental Sciences, College of Agriculture, Engineering and Science, University of KwaZulu-NatalPietermaritzburg, South Africa; ^2^Directorate of Research and Training, Plant Production Research, Ministry of Agriculture, Water and ForestryWindhoek, Namibia; ^3^African Centre for Crop Improvement, University of KwaZulu-NatalPietermaritzburg, South Africa

**Keywords:** cowpea, gamma radiation, mutation breeding, mutants, legume improvement

## Abstract

Cowpea (*Vigna unguiculata* [L.] Walp.) yields are considerably low in Namibia due to lack of improved varieties and biotic and abiotic stresses, notably, recurrent drought. Thus, genetic improvement in cowpea aims to develop cultivars with improved grain yield and tolerance to abiotic and biotic stress factors. The objective of this study was to identify agronomically desirable cowpea genotypes after mutagenesis using gamma irradiation. Seeds of three traditional cowpea varieties widely grown in Namibia including Nakare (IT81D-985), Shindimba (IT89KD-245-1), and Bira (IT87D-453-2) were gamma irradiated with varied doses and desirable mutants were selected from M_2_ through M_6_ generations. Substantial genetic variability was detected among cowpea genotypes after mutagenesis across generations including in flowering ability, maturity, flower and seed colors and grain yields. Ten phenotypically and agronomically stable novel mutants were isolated at the M_6_ each from the genetic background of the above three varieties. The selected promising mutants’ lines are recommended for adaptability and stability tests across representative agro-ecologies for large-scale production or breeding in Namibia or similar environments. The novel cowpea genotypes selected through the study are valuable genetic resources for genetic enhancement and breeding.

## Introduction

Cowpea (*Vigna unguiculata* L. Walp.) is a leguminous species used as food, forage, and vegetable crop mainly in the tropics (Steele, 1972). The grains are an excellent source of food and feed; a vital nutrient for healthy growth both for humans and livestock. The leaves, green pods, and grains are consumed as a dietary source of protein (Ghaly and Alkoaik, 2010). The cowpea grain contains 23% protein and 57% carbohydrate, and the leaves contain 27–34% of proteins. The crop originated and domesticated in Southern Africa, which was later spread to east and West Africa and Asia (International Institute for Tropical Agriculture [IITA], 2004). In semi-arid West and Central Africa, it is consumed as a pulse where it supplements the daily diet (Bressani, 1985). Thus, cowpea production remains the most prominent food legume cultivated by farmers majorly in most sub-Saharan African countries. The main reasons being the natural ability of the crop to withstand moderate episodes of drought and its adaptation to grow in nutrient limited soils. Cowpea is also able to fix atmospheric nitrogen in marginal soils where farmers are unable to adequately fertilize their crops due to unaffordability or inaccessibility (Steele, 1972). Accounts indicate that greater than 16,000 genotypes of cowpea are registered in trust for the World Bank by the International Institute of Tropical Agriculture, (IITA) Ibadan, Nigeria. Such a huge genotype bank is believed to provide a wide range of information on the agronomy and potential benefits of the crop.

The southern African region is reportedly considered the centre of diversity of *V. unguiculata* which includes Namibia, Botswana, Zambia, Zimbabwe, Mozambique, and the Republic of South Africa (Ng and Marachel, 1985). In Namibia, cowpea is the second most important crop next to pearl millet. Nearly, 95% of the smallholder farmers in the northern part of the country grow cowpea for food security and/or livelihoods. However, cowpea yields of the available cultivars are considerably low (250–350 kg/ha) predominantly due to lack of improved varieties and biotic and abiotic stresses notably recurrent severe drought. Hence, genetic improvement in cowpea requires systematic breeding and development of genotypes associated with higher yielding capacity and drought resilience.

Genetic variation is the basis for plant breeding programs. Most conventional crop improvement programs rely on natural genetic variation present among germplasm pools (Ceccarelli and Grando, 2007). Mutations can be induced in various ways, such as exposure of plant propagules, including seeds, tissues, and organs, to physical and chemical mutagens (Mba et al., 2010). Induced mutagenesis has the potential to create genetic variation for genetic enhancement and breeding in a relatively shorter time unlike natural mutation or controlled crosses of especially unrelated parents (Singh et al., 2006; Wani, 2006; Tulmann Neto et al., 2011). Gnanamurthy et al. (2012) reported that induced mutations have been successfully used in breeding of seed propagated crops since 1940s. The Mutant Varieties Database (MVD) of FAO (Food and Agriculture Organisation of the United Nations) and the International Atomic Energy Agency (IAEA) maintained a list of 2,252 crop cultivars developed through artificial mutations (Nielen, 2004). These cultivars were released across 59 countries worldwide, mainly in the continental Asia (1,142 cultivars), Europe (847), and North America (160) (Maluszynski, 2001; Maluszynski et al., 2009). Studies indicate that induced mutagenesis has successfully modified several plant traits such as plant height, maturity, seed shattering resistance, disease resistance, oil quality and quantity, malting quality, size and quality of starch granules of cowpea (Goyal and Khan, 2010; Singh et al., 2013).

In South Africa, cowpea mutants were developed through selections from the M_2_ to M_4_ generations. These included the drought tolerant mutants such as 447, 217, and 346, and mutants such as 447, MA2, and 217 isolated for their high yielding ability under well-watered conditions (De Ronde and Spreeth, 2007). Furthermore, early maturing cowpea mutants with leaflets containing tendrils, broad leaves, and light green pods were developed through gamma irradiation in Nigeria (Adekola and Oluleye, 2007). The use of gamma irradiation at different doses has been reported to change the proximate and anti-nutritive compositions in pulses (Udensi et al., 2012). Some varieties of groundnut were developed in Congo through gamma irradiation (Tshilenge-Lukanda et al., 2012). Wani (2006) reported a significant increase in the mean values of the fertile branches per plant, pods per plant and seed yield per plant (SYP) in mutant varieties of mungbean (*Vigna radiata* [L.] Wilczek) derived through gamma irradiation.

In light of this, a collaborative research was developed in 2009 between the Namibian Government and the IAEA under Technical Cooperation project on induced mutation breeding using Gamma irradiation. This created a platform for pre-breeding and breeding of high yielding, drought tolerant and insect pest resistant genotypes of cowpea. Gamma irradiation was recommended by the Namibian Radiation Regulatory Authority as an alternative option to create new crop genotypes in a short period of time without any negative impact to the environment.

Therefore, the objective of this study was to identify desirable cowpea genotypes after gamma irradiation of three traditional cowpea varieties widely grown in Namibia including Nakare (IT81D-985), Shindimba (IT89KD-245-1), and Bira (IT87D-453-2) through continuous selections from M_2_ through M_6_ generations.

## Materials and Methods

### Plant Material and Gamma Irradiation

Three cowpea genotypes widely grown in Namibia, namely, Nakare (IT81D-985), Shindimba (IT89KD-245-1) and Bira (IT87D-453-2) were obtained from Likorerere Farmers Co-operatives at Kavango Region, Namibia. The seeds were irradiated at the International Atomic Energy Agency (IAEA), Agriculture and Biotechnology Laboratory, A-2444 Seibersdorf, Austria using a CO60 source Gammacell Model No. 220. Various doses were used to establish the optimum irradiation level that can achieve optimum mutation frequency with least possible and unintended damage. The three varieties were gamma irradiated as follows: Bira [0, 75, 150, 300, 450, and 600 Gy], Nakare [0, 100, 150, 200, 250, and 300 Gy] and Shindimba [0, 100, 150, 200, 300, and 400 Gy]. Preliminary tests showed that the three varieties differed in their optimal requirement of irradiation doses and was used as the bases for using different doses for each genotype (Horn and Shimelis, 2013). The 0 Gy dose served as a comparative control.

### Study Sites, Experimental Design, and Field Establishment

A series of selection experiments were conducted at three different sites; namely Mannheim, Bagani, and Omahenene. Mannheim Research Station is located in Oshikoto region along the north central of Namibia and it is situated at an altitude of 1234 m above sea level (masl). Bagani Research Station is located at (1007 masl) north east in the Kavango East region, whereas Omahenene research station is situated in the Omusati Region in North-Western Namibia at altitude of 1109 masl. In general, climatic, biological conditions of the selection sites vary considerably. Physicochemical properties of the sites are provided in **Table 1**. The M_1_ and M_2_ generations were evaluated at Mannheim Research Station during the 2009/2010 and 2010/2011 seasons, respectively. The M_3_ generations were established at Bagani research station during the 2011/2012 season. The M_4_ and M_5_ were established at Omahenene Research Station in 2012/2013 and 2013/2014 season, respectively.

**Table 1:** Physicochemical properties of soils at Mannheim, Bagani, and Omahenene research sites in Namibia.

Sample/parameter	Research Station (study site)		
	Mannheim	Bagani	Omahenene
Soil pH	7.87	7.5	8.2
Total Nitrogen%	0.06	0.06	0.05
Organic carbon%	0.38	0.48	0.60
Phosphorus (ppm)	18	58.2	14
Potassium me%	0.17	0.9	0.99
Calcium me %	1.6	1.3	1.38
Magnesium me%	4.74	1.7	4.80
Manganese me%	0.05	0.18	0.17
Copper (ppm)	0.6	0.6	0.5
Iron (ppm)	0.5	0.7	0.5
Zinc (ppm)	0.6	0.5	0.4
Sodium %	0.10	0.09	0.07
EC mS/cm	0.29	0.18	0.36

*ppm, part per million; me, milliequivalent; EC, electrical conductivity.*

**FIGURE 1:**
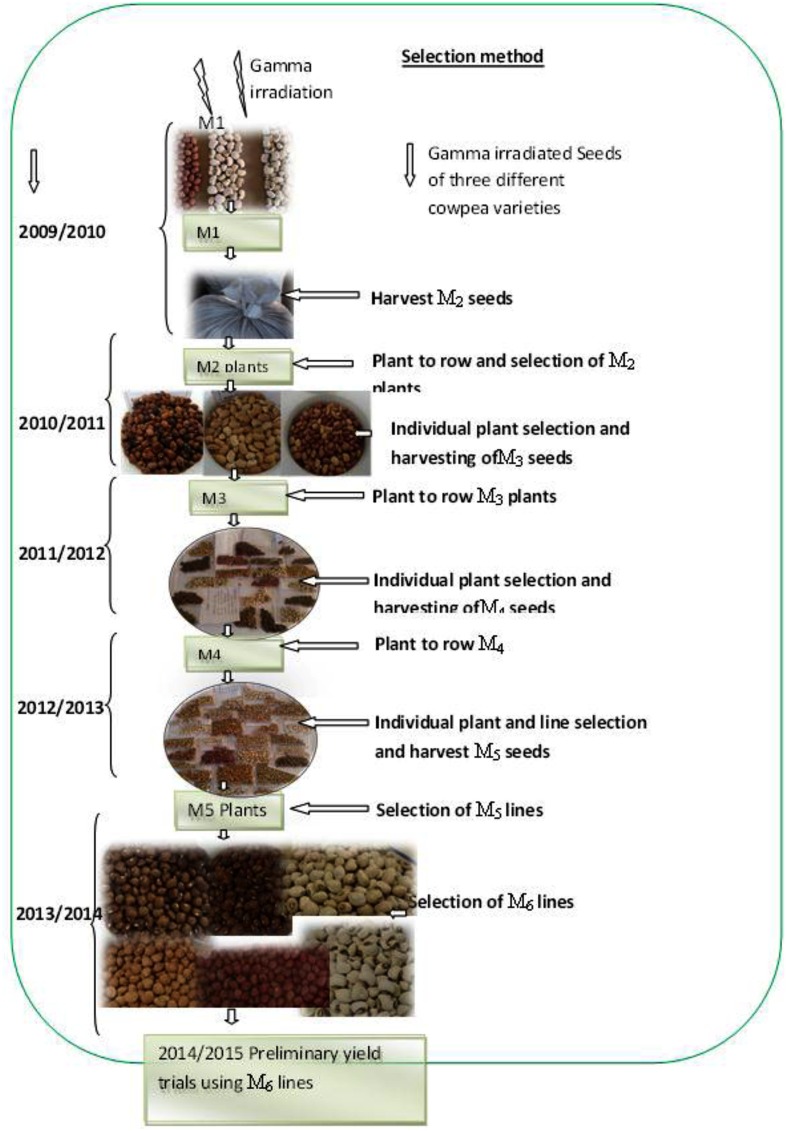
**Illustration of the selection methods during 2009–2014**.

Plots were arranged in a randomized complete block design using two replications. Plants were established using intra-row spacing of 20 cm and inter-row spacing of 75 cm. Seedlings were thinned to one plant per hill after 2 weeks from planting. Weeds were controlled manually. Planting of the M_1_ seeds was done under normal growing conditions with supplemental irrigation during dry spell. Each row of the M_1_ generation contained 26 individuals, making a total of 104 plants per irradiation dose. At harvest the M_2_ seeds were bulked in separate bags according to irradiation doses (**Figure 1**). During the M_2_ to M_5_ generations’ variable number of individual plants ranging from 50 to 100 per irradiation dose were assayed for qualitative and quantitative observations.

### Selection Procedure and Data Collection

The selection procedure was undertaken based on methods adapted from Maluszynski et al. (2009). The selection procedure used in the study is illustrated in **Figure 1**. The irradiated seeds (M_1_) were planted in the field at Mannheim research station under standard cultural practices. All the pods, from the M_1_ plants that survived were harvested and bulked according to their respective radiation doses and genotypes. Consequently, the harvested M_2_ seeds were planted in the field at Mannheim as M_2_ population during 2010/2011 season in the form of progeny rows for individual plant selection and to develop the M_3_ seeds. The M_3_ seed from selected M_2_ plants were planted at Omahenene and Bagani Research Station during 2011/2012 for evaluation. The M_3_ plants at both sites were evaluated in the field using morphological and agronomical attributes. Pods from selected M_3_ plants were harvested. During 2012/2013, the M_4_ seeds obtained from the selected M_3_ population were planted at Omahenene Research Station as single-plant progenies and segregants were selected with desired traits. During 2013/2014 the M_5_ seeds obtained from the selected M_4_ population were planted at Omahenene Research Station as single-plant progenies and selection were made toward desired trait on single plant basis. Uniform, non-segregating mutant progenies, were bulked at this stage to hasten the breeding cycle. During 2014/2015 the M_6_ generation was evaluated at Omahenene, Bagani, and Mannheim using suitable lines selected for seed yield and related traits.

### Data Collection and Analysis

Both quantitative and qualitative data were collected during evaluations from the M_2_ to M_5_ generations. The data collected included: days to 50% germination (DG), percent seed emergence (ES%), number of abnormal individuals or visual phenotype mutants (ABN), total number of surviving plants per plot (TNP), number of main branches (NMB) averaged over 10 randomly selected and tagged plants, days to 50% flowering (DTF), days to 50% pod setting (DPS), days to 50% maturity(DMT), number of pods per plant (NPP) averaged over five pods per selected plant, pod length (PL) expressed in cm and averaged over five pods per plant, pod weight per plant (PW) in gram, number of seeds per pod (NSP) averaged over five pods per plant, 100 seed weight (HSW) in gram and SYP in gram. The qualitative data collected included variation in flower color (FC) and seed color (SC) during the M_1_ and M_2_ generations. Additional qualitative data such as, pod shape (PS), pod color (PC), seed coat texture (SCT), and growth habit (GH) were collected from M_2_ to M_5_ generations. Data were analyzed and descriptive statistics summarized using the SAS statistical program (SAS, 2002).

## Results

### Phenotypic Characterization of Mutants

#### Qualitative and Quantitative Traits at M_1_ and M_2_

**FIGURE 2:**
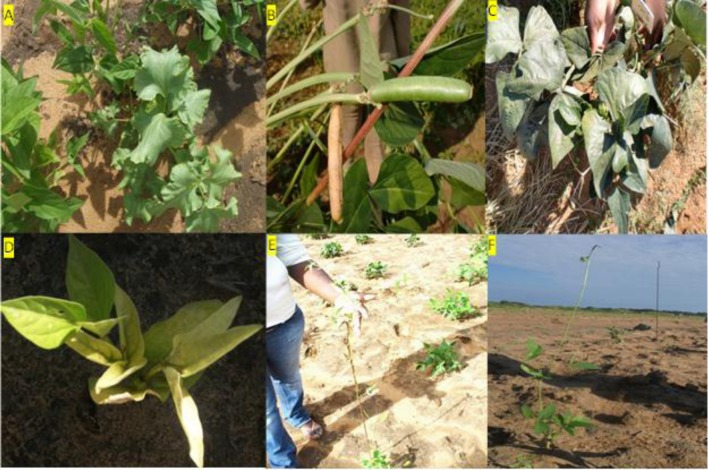
**Some common abnormalities at M_3_ observed at Bagani Research Station. (A)** spinach-like leaves, **(B)** Short-pods, **(C)** broad-dark leaves, **(D)** chlorophyll mutant, -single stem **(E,F)** observed at Omahenene research Station.

**FIGURE 3:**
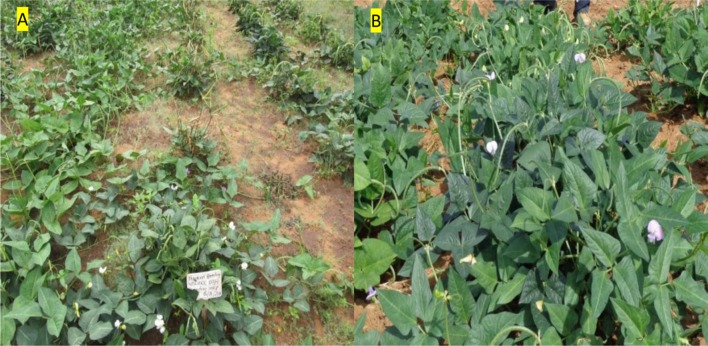
**Variation in flower color **(A)** white flower color, **(B)** purple flower and field plant stands of M_5_ Nakare mutants observed at Omahenene Research Station in Namibia**.

**FIGURE 4:**
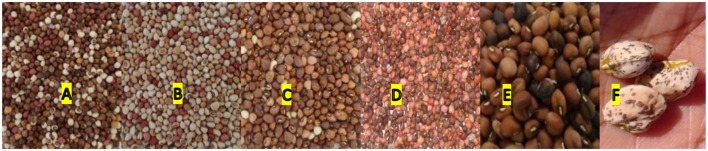
**Different M_3_ seed colors **(A–F)** observed among all mutants at all locations**.

During the M_1_ and M_2_ generations the percentage field establishment (ES) ranged between 79 to 89%, respectively (**Table 2**). Nakare and Shindimba mutants had ES of 0% at irradiation does of 250, 300, and 400 Gy. Phenotypic abnormalities such as albinism, leaf deformity, single stem, seedless pods or short pod sizes were invariably observed at the following doses and genotypes: 450 and 600 Gy (Bira); 150 and 200 Gy (Nakare); and 100, 150, and 200 Gy (Shindimba) (**Figure 2**). Segregation of FC (white and purple) were observed at the M_2_ with the following doses and genotypes: 300, 450, and 600 Gy (Bira), 100 and 200 Gy (Nakare), and 100, 150, and 200 Gy (Shindimba) (**Figure 3**)_._ SC variations were observed during the M_2_ (**Figure 4**)_._ White, brown, red, and cream SC were common in Bira mutants across all irradiation doses. In addition to these Nakare and Shindimba had speckled, chocolate, light brown, black, mixed and dark brown SC when subjected to irradiation doses of 100, 150, and 200 Gy (**Table 2** and **Figure 4**). Bira mutants displayed relatively high seed yields varying from 98 to 200 g/plant at 0 and 600 Gy, respectively (**Table 2**).

**Table 2:** Phenotypic characteristics of mutants observed during the first two seasons 2009/2010 and 2010/2011 at Mannheim Research Station.

Variety	M_1_ (2009/2010)						M_2_ (210/2011)				
	Dose (Gy)	ES%	ABN	FC	SC	SYP	ES%	ABN	FC	SC	SYP
Bira	0	89	0	2	3	2.9	99	0	2	3	98
	75	80	0	2	3	2.9	88	0	2	1,2,3,4	150
	150	87	0	2	3	3.1	89	0	2	1,2,3,4	162
	300	82	1	2	3	2.0	90	1,2,3	1,2	1,2,3,4	160
	450	81	1,2	2	3	1.6	93	1,2,3	1,2	1,2,3,4	158
	600	79	1,2,3,4,5	2	3	1.1	97	1,2,3,5	1,2	1,2,3,4	200
Nakare	0	86	0	1	1	1.6	89	0	1	1	90
	100	49	0	1	1	1.3	88	0	1,2	1,2,4,5,6	75
	150	46	1,2,3,4	1	1	0.3	86	1,2,3	1,2	1,2,4,5,6	81
	200	8	1,2,3,4	1	1	0.5	80	1,2,3,4,5	1,2	1,2,4,5,6	71
	250	0	N/A	N/A	N/A	0.0	N/A	N/A	N/A	N/A	N/A
	300	0	N/A	N/A	N/A	0.0	N/A	N/A	N/A	N/A	N/A
Shindimba	0	88	0	1	1	1.9	95	0	1	1	70
	100	35	1,2,3,4	1	1	1.4	86	1,2,3	1,2	1,2,4,5,6	66
	150	37	1,2,3,4	1	1	0.8	93	1,2,3	1,2	1,2,4,5,6	65
	200	18	1,2,3,4	1	1	0.1	90	1,2,3,4,5	1,2	1,2,4,5,6	60
	300	0	N/A	N/A	N/A	N/A	N/A	N/A	N/A	N/A	N/A
	400	0	N/A	N/A	N/A	N/A	N/A	N/A	N/A	N/A	N/A

*ES%, establishment in %; ABN, abnormalities observed, where 0 = normal, 1 = albino, 2 = leafy type, 3 = upright single stem, 4 = seedless pods, and 5 = short pods; FC, flower color, where 1 = white, 2 = purple. SC, seed color; where 1 = white, 2 = brown, 3 = red, 4 = cream, 5 = speckled, 6 = chocolate, 7 = light brown, 8 = black, 9 = mixed, 10 = dark brown. SYP, seed yield (gram/plant).*

#### Qualitative Traits Evaluated during the M_3_ to the M_5_

**FIGURE 5:**
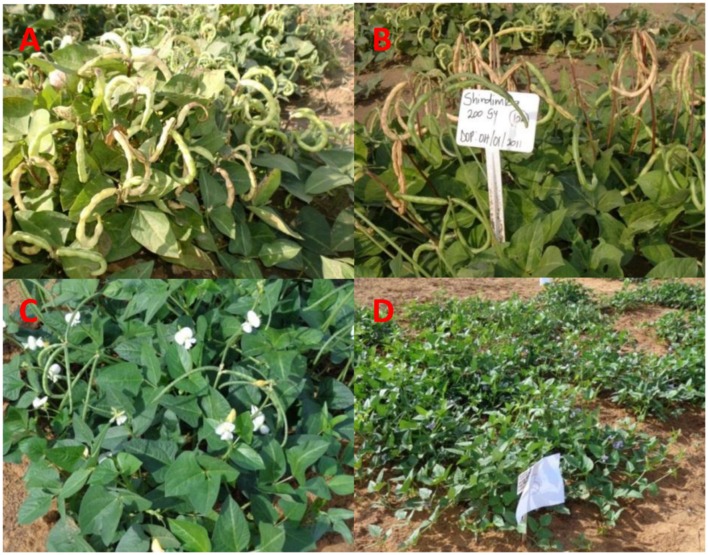
**Variation among Shindimba mutant lines. (A)** coiled pods, **(B)** semi-coiled pods observed at Mannheim during the M_2_ generation, **(C)** white flower with semi-coiled pods, and **(D)** Purple flowers observed at Omahenene during the M_5_ generation.

Variable number of individual plants was available for selection during M_3_ to M_5_ generations, because of the strength of irradiation treatment and segregation. The following doses allowed successful selections of mutants during the M_3_ to M_5_: 300, 450, and 600 Gy (Bira), 100 and 150 Gy (Nakare), and 100 and 200 Gy (Shindimba). Surviving and phenotypically stable individuals were advanced at each selection generation at Omahenene and Bagani Research Stations. Qualitative traits had limited variation during M_3_ to M_5_ (**Table 3**). Bira mutants displayed purple FC irrespective of doses and test generations, while Nakare and Shindimba segregated for white and purple FC (**Figures 3** and **5**). Both Bira and Nakare mutants had straight PS similar to the controls. However, Shindimba segregants had straight and coiled pod types (**Figure 5**). Variable SCs including white, brown, red, cream, speckled, chocolate, light and dark brown, black and mixed were observed during the M_3_ to M_5_. Bira mutants had smooth SCT, while Nakare and Shindimba had mainly rough and smooth seed texture. Bushy, erect and spreading GHs were detected during the M_3_ to M_5_ (**Figures 3** and **5**).

**Table 3:** Qualitative traits observed among the mutant lines at the M_3_, M_4_, and M_5_ at Omahenene and Bagani Research Stations.

Genotype	Dose (Gy)	FC	PS	PC	SC	SCT	GH	PI
Bira	0	2	1	1	3	1	3	1
	300	2	1	1	1,2,3,4	1	1,2,3	1
	450	2	1	1	1,2,3,4	1	1,2,3	1
	600	2	1	1	1,2,3,4,5,6,7,8	1	1,2,3	1
Nakare	0	1	1	1	1	2	2	1
	100	1,2	1	1	1,2,3,6,7,9,10	1,2	1,2	1
	150	1,2	1	1	1,2,3,6,7,9,10	1,2	1,2	1
Shindimba	0	1	2	1	1	1,2	2	1
	100	1,2	1,2	1	1,2,3,7,9,10	1,2	1,2	1
	200	1,2	1,2	1	1,2,3, 7,9,10	1,2	1,2	1

*FC, flower color, where 1 = white, 2 = purple; PS, pod shape, where 1 = straight, 2 = coiled or curved; PC, pod color, where 1 = cream; SC = seed color, where 1 = white, 2 = brown, 3 = red, 4 = cream, 5 = speckled, 6 = chocolate, 7 = light brown, 8 = black, 9 = mixed, 10 = dark brown; SCT, seed coat texture, where 1 = smooth and 2 = rough; GH, growth habit, where 1 = bushy, 2 = erect and 3 = spreading; PI, pest infestation, where 0 = none, 1 = mild, and 2 = severe.*

#### Quantitative Traits Observed from M_3_ to M_5_

Quantitative traits of agronomic importance were measured during the M_3_ to M_5_ (**Table 4**). The percent seed emrgence (ES%) reduced significantly with increased irradiation dose. Maximum seed germination was achieved 3 days after planting irrespective of irradiation doses (**Tables 4–6**). Shindimba mutants relatively flowered early (40 days) at the M_3_ (**Table 4**).

**Table 4:** Quantitative characteristics of M_3_ cowpea mutant lines irradiated at different gamma radiation doses (Gy) in relation to their parental lines/control (Gy 0) observed at Bagani Research Station during 2011/2012 season.

Variety	Dose (Gy)	TNP	ES%		DG	DTF	DPS	DMT	NPP	PL	PW	NSP	HSW	SYP
Bira	0	330	100	Min	3	47	51	57	3.0	13.0	7.0	7.6	13.0	3.0
				Max	3	58	63	74	57.0	20.6	136	18.0	18.0	94.0
				Mean	3	50	55	64	28.6	15.1	64.9	14.5	15.1	40.9
	
	300	330	90.6	Min	3	40	49	52	4.0	10.4	3.0	9.0	9.0	1.0
				Max	3	80	86	92	5439.0	20.0	4003.0	19.0	25.0	3500.0
				Mean	3	52	58	63	322.6	16.9	211.9	10.8	14.4	136.6
	
	450	330	60.6	Min	N/A	N/A	N/A	N/A	N/A	N/A	N/A	N/A	N/A	N/A
				Max	N/A	N/A	N/A	N/A	N/A	N/A	N/A	N/A	N/A	N/A
				Mean	N/A	N/A	N/A	N/A	N/A	N/A	N/A	N/A	N/A	N/A
		330	93	Min	3	46	50	57	1.0	5.0	1.0	1.8	4.0	1.0
	
	600			Max	3	59	63	74	120.0	21.3	218	18.6	21.0	142.0
				Mean	3	50	54	63	16.4.0	14.9	36.6	12.2	14.9	22.0

Nakare		330	45.5	Min	3	11	25	32	11.0	14.2	11.5	5.0	16.2	1.3
	0			Max	3	35	34	43	85.0	19.6	220.6	12.0	30.5	138.8
				Mean	3	20	29	36	51.2	15.8	106.1	7.7	25.3	59.2
		330	78.5	Min	3	10	25	32	3.0	6.8	2.8	2.0	11.4	0.7
	
	100			Max	3	37	62	65	125.0	23.5	327.8	17.2	32.1	191.2
				Mean	3	19	29	37	32.3	16.7	65.2	9.0	23.5	32.9
		330	60.3	Min	3	43	45	46	3.0	5.6	3.0	1.8	6.1	N/A
	
	150			Max	3	78	95	96	172.0	22.7	360.8	18.4	109	N/A
				Mean	3	52	57	66	39.0	14.2	67.6	10.2	15.8	N/A

Shindimba		330	69.7	Min	3	49	55	60	3.0	10.0	3.0	6.3	15.0	2.0
	0			Max	3	69	75	83	38.0	19.0	110.0	12.0	19.0	62.0
				Mean	3	57	62	70	23.0	16.0	44.7	9.5	17.5	22.2
		330	36	Min	3	49	54	60	3.0	10.0	3.0	5.8	14.0	1.0
	
	100			Max	3	84	90	98	40.0	21.0	110.0	16.6	25.0	118.0
				Mean	3	59	63	71	21.0	16.0	35.4	9.5	17.7	17.7
		330	69	Min	3	15	47	N/A	2.0	1.3	1.1	3.0	8.1	N/A
	
	200			Max	3	76	98	N/A	109.0	21.9	220.2	15.4	48.7	N/A
				Mean	3	62	68	N/A	32.0	14.6	55.5	7.9	20.8	N/A

*TNP, total number of plants per plot; ES%, percentage establishment; DG, days to 50% germination; DTF, days to 50% flowering; DPS, days to 50% pod setting; DMT, days to 50% maturity; NPP, number of pods per plant; PL, pod length (in cm); PW, pod weight (gram), NSP, number of seeds per pod, HSW, 100 seed weight (gram), SYP, seed yield (gram/per plant), and N/A, data not available.*

At the M_4_ a relatively shorter days to flowering (44 days) was recorded at 300 Gy (**Table 5**). Contrastingly, the number of days to flowering was 37 days at the M_5_ at using 600 Gy (**Table 6**). Nakare derived mutants flowered relatively earlier (10 days) at 100 Gy at the M_3_ (**Table 4**). At the M_5_ Nakare mutants recorded a minimum of 61 days to flowering at 0 and 150 Gy (**Table 5**). At the M_3_, Shindimba mutants displayed a minimum of 15 and a maximum of 84 days to flowering at 200 and 100 Gy, respectively (**Table 4**). Nakare mutants recorded the lower days (25) for pod setting (DPS) at the M_3_when using 100 Gy. Comparatively, the higher number of DPS (98 days) was measured in Shindimba at 200 Gy. At the M_4_ a minimum DPS of 48 days was recorded for Bira derivatives at 300 Gy. A maximum DPS of 86 days was recorded for Bira mutants at 400 Gy, Nakare at 100 and 150 Gy and Shindimba at 100 and 200 Gy (**Table 5**). At the M_5_, Nakare mutants recorded the lower DPS (41 days) at 100 Gy, while Bira genotypes had the higher DPS of 88 days at 300 Gy, **Table 6**). During the M3, Nakare mutants matured 32 days after planting at 100 Gy. At the same dose rate Shindimba displayed late maturity (98 days) at the M_3_ (**Table 4**)_._ During the M_4_ Bira mutants matured earlier (54 days) at 450 Gy. Delayed maturity (115 days) were recorded for Nakare at 150 Gy and Shindimba at 100 and 200 Gy (**Table 5**). At the M_5_ Bira measured early maturity (62 days) with the highest dose of 600 Gy. Interestingly, this genotype matured late (115 days) when subjected to irradiation dose of 300 Gy (**Table 6**). Nevertheless, Bira recorded lower NPP (1 pod/plant) at 600 Gy and higher (5 pods/plant) when irradiated at 300 Gy (**Table 4**). At the M_4_, 1 pod/plant was recorded for Bira at 450 and 600 Gy and Shindimba at 200 Gy (**Table 5**).

**Table 5:** Quantitative characteristics of M_4_ cowpea mutant lines irradiated at different gamma radiation doses (Gy) in relation to their parental lines/control (Gy 0) observed at Omahenene Research Station during 2012/2013 season.

Variety	Dose (Gy)	TNP	ES%		DG	DTF	DPS	DMT	NPP	PL	PW	NSP	HSW	SYP
Bira	0	330	69.7	Min	3	45	49	59	8.0	14.0	13.0	5.0	10.0	6.0
				Max	3	48	53	72	88.0	20.0	231.0	20.0	16.0	187.0
				Mean	3	46	50	68	31.0	17.7	86.2	14.2	13.0	53.4
	
	300	330	55.0	Min	3	44	48	66	2.0	9.0	4.0	6.0	5.0	1.0
				Max	3	51	55	74	97.0	21.0	325.0	18.0	79.0	287.0
				Mean	3	46	50	69	31.0	16.8	79.6	14.3	13.0	52.9
	
	450	330	85.7	Min	3	45	49	54	1.0	10.0	2.0	4.0	9.0	1.0
				Max	3	81	86	90	127.0	16.0	330.0	20.0	115.0	195.0
				Mean	3	49	54	60	26.0	15.8	50.0	17.0	15.0	30.0
	
	600	330	85.0	Min	3	46	50	57	1.0	6.0	1.0	2.0	4.0	1.0
				Max	3	59	63	74	124.	22.0	224.0	19.0	21.0	160.0
				Mean	3	50	55	63	18.4	16.3	41.6	13.0	15.1	25.1

Nakare	0	330	42.0	Min	3	61	66	96	5.0	11.0	9.0	4.0	17.0	6.0
				Max	3	74	78	110	32.0	19.0	85.0	17.0	26.0	62.0
				Mean	3	70	75	104	14.0	15.1	29.8	9.8	22.8	22.8
	
	100	330	56.0	Min	3	61	66	86	2.0	10.0	4.0	5.0	5.0	2.0
				Max	3	78	86	113	70.0	21.0	227.0	14.0	59.0	199.0
				Mean	3	71	76	103	15.0	15.5	35.3	10.0	21.4	26.7
	
	150	330	88.8	Min	3	61	67	86	2.0	8.0	3.0	3.0	3.0	1.0
				Max	3	79	86	115	85.0	26.0	287.0	18.0	40.0	131.0
				Mean	3	71	76	103	21.9	16.5	48.0	11.0	17.3	33.5

Shindimba	0	330	93.9	Min	3	42	68	72	7.0	7.0	7.0	3.0	12.0	3.0
				Max	3	75	78	85	44.0	29.0	123.0	13.0	30.0	91.0
				Mean	3	71	72	76	20.0	13.0	33.0	7.4	20.1	25.1
	
	100	330	82.4	Min	3	42	66	87	2.0	7.0	3.0	3.0	10.0	2.0
				Max	3	80	86	115	63.0	23.0	130.0	19.0	30.0	91.0
				Mean	3	71	76	104	16.7	13.8	27.7	8.1	19.8	20.9
	
	200	330	68.5	Min	3	62	66	94	1.0	9.0	3.0	3.0	6.0	2.0
				Max	3	80	86	115	59.0	31.0	123.0	16.0	30.0	91.0
				Mean	3	72	76	104	15.5	13.0	26.5	8.1	18.4	19.3

*TNP, total number of plants per plot; ES%, percentage establishment; DG, days to 50% germination; DTF, days to 50% flowering; DPS, days to 50% pod setting; DMT, days to 50% maturity; NPP, number of pods per plant; PL, pod length (in cm); PW, pod weight (gram), NSP, number of seeds per pod, HSW, 100 seed weight (gram), SYP, seed yield (gram/per plant).*

**Table 6:** Quantitative characteristics of M_5_ cowpea mutant lines irradiated at different gamma radiation doses (Gy) in relation to their parental lines/control observed at Omahenene Research Station during 2013/2014 season.

Variety	Dose (Gy)	TNP	ES%		DG	DTF	DPS	DMT	NPP	PL	PW	NSP	HSW	SYP
Bira	0	330	97.0	Min	3	68.0	73.0	98.0	7.0	16.0	15.0	6.0	9.0	10.0
				Max	3	83.0	88.0	115.0	66.0	27.0	155.0	18.0	16.0	115.0
				Mean	3	74.0	78.0	102.0	40.7	21.0	88.9	14.3	12.9	61.9
	
	300	330	77.6	Min	3	64.0	69.0	98.0	3.0	13.0	6.0	5.0	4.0	3.0
				Max	3	83.0	88.0	115.0	150.0	30.0	325.0	20.0	29.3	213.0
				Mean	3	73.0	78.0	102.0	30.9	21.0	66.6	14.2	12.7	47.0
	
	450	330	85.8	Min	3	42.0	46.0	66.0	2.0	13.0	6.0	5.0	11.0	3.0
				Max	3	58.0	69.0	76.0	233.0	20.0	659.0	20.0	171.0	570.0
				Mean	3	47.0	51.0	70.0	31.6	17.7	81.5	14.9	16.2	60.0
	
	600	330	78.5	Min	3	37.0	42.0	62.0	1.0	9.0	1.0	3.0	4.0	2.0
				Max	3	56.0	61.0	81.0	78.0	27.0	276.0	18.0	19.0	157.0
				Mean	3	46.0	50.3	68.6	19.9	16.3	43.4	13.2	12.4	28.1

Nakare	0	330	42.0	Min	3	41.0	45.0	65.0	47.0	16.0	76.0	13.0	1.0	51.0
				Max	3	53.0	57.0	79.0	46.0	21.0	72.0	12.0	298.0	51.0
				Mean	3	47.0	50.8	69.0	45.0	16.8	70.8	12.3	62.8	50.5
	
	100	330	56.4	Min	3	37.0	41.0	58.0	1.0	9.0	1.0	1.0	1.0	1.0
				Max	3	57.0	60.0	80.0	144.0	23.0	375.0	18.0	81.0	298.0
				Mean	3	46.0	49.5	66.0	39.0	17.7	86.8	12.0	18.1	62.8
	
	150	330	59.7	Min	3	42.0	46.0	64.0	1.0	10.0	3.0	5.0	6.0	2.0
				Max	3	53.0	57.0	73.0	110.0	28.0	317.0	20.0	82.0	209.0
				Mean	3	46.0	50.0	68.0	29.0	21.0	70.6	12.5	18.4	45.6

Shindimba	0	330	93.9	Min	3	50.0	54.0	66.0	2.0	8.0	5.0	3.0	2.0	2.0
				Max	3	55.0	59.0	73.0	88.0	20.0	127.0	11.0	29.0	90.0
				Mean	3	52.3	56.0	69.0	33.8	13.6	64.0	8.0	22.0	42.0
	
	100	330	86.7	Min	3	42.0	46.0	60.0	1.0	8.0	1.0	1.0	2.0	1.0
				Max	3	67.0	70.0	89.0	122.0	25.0	392.0	18.0	29.0	208.0
				Mean	3	50.5	54.2	70.0	35.5	14.0	75.0	8.0	21.5	50.0
	
	200	330	83.6	Min	3	44.0	48.0	65.0	1.0	7.0	1.0	1.0	6.0	1.0
				Max	3	67.0	71.0	91.0	89.0	29.0	193.0	18.0	25.0	93.0
				Mean	3	52.0	56.0	73.0	20.0	16.0	39.0	9.0	16.0	25.0

*TNP, total number of plants per plot; ES%, percentage establishment; DG, days to 50% germination; DTF, days to 50% flowering; DPS, days to 50% pod setting; DMT, days to 50% maturity; NPP, number of pods per plant; PL, pod length (in cm); PW, pod weight (gram), NSP, number of seeds per pod, HSW, 100 seed weight (gram), SYP, seed yield (gram/per plant).*

At the M_3_ the longer pod size measured at 23.5 cm was recorded for Nakare at 100 Gy (**Table 4**). At the M_4_, Shindimba mutants resulted from 200 Gy measured longer pod size of 31 cm (**Table 5**). Bira mutants induced with 300 Gy produced longer pod size (30 cm) (**Table 6**). Relatively heavier pod size (4003 g/plant) was recorded for Bira at 300 Gy (**Table 4**). At the M4, Bira had pod size measured at 325 g/plant at 300 Gy. Notably this genotype had reduced pod weight (1 g/plant) at the highest irradiation dose (**Table 5**).

The NSP varied significantly between irradiation doses and genotypes. At the M_3_, the highest number of seeds of 18.6/pod was recorded for Bira at 600 Gy and Nakare 150 Gy (**Table 4**). At the M_4_ 19 seeds/pod was achieved in the mutants of Bira at 600 Gy and Shindimba at 100 Gy. At the M_5,_ mutants of Bira derived from 300 and 450 Gy and Nakare 150 Gy recorded 20 seeds/pod, the highest in this trial (**Table 5**). Hundred seed weight (HSW) at M_3_ was relatively heavier measured at 109 g for Nakare mutants derived from 150 Gy (**Table 4**). At the M_4_ the higher HSW (115 g) was recorded for Bira at 450 Gy (**Table 5**). During the M_5_ Bira displayed higher HSW of 171 g at 450 Gy (**Table 6**). High seed yield per plant is an economic trait for cowpea growers. At M_3_, higher seed yield of 3500 g per plant was recorded for Bira mutants derived from the mutagenic treatment of 300 Gy (**Table 4**). During the M_4_ generation Bira and Nakare mutants derived from 300 Gy and 100 Gy had a relatively higher seed yields of 287 and 199 g/plant, in that order (**Table 5**). At the M_5_ generation Bira mutants yielded 570 g/plant, while Nakare had 298 g/plant when subjected to 450 Gy and 100 Gy, respectively (**Table 6**).

## Discussion

The present study revealed the important roles of induced mutations in cowpea breeding. It was evident from this study that increased Gy doses above 150 Gy can be lethal for the cowpea breeding line such as Nakare, while a dose above 200 Gy is lethal for the breeding line Shindimba. Other authors have reported the negative effects of increased mutagenic doses affecting various crops’ establishment and survival for breeding (Mba et al., 2009).

The present study showed the presence of clear phenotypic differences among the tested mutant lines when compared to their respective controls. Overall, increased irradiation dose was associated with visual phenotypic mutants. Mutants displayed visual phenotypic differences including chlorophyll, leaf, upright single stem, pod, and seed during the M_2_ to M_5_ generations. Chlorophyll mutants observed were plants with yellow and striped leaves, albinos or yellow to pale leaf and stem pigmentations. Virescence mutants showed broad pale green leaf with its margin resembling a spinach leaf (**Figure 2**). According to Girija and Dhanavel (2009) and Maluszynski et al. (2009), the appearance of chlorophyll defects is a good indicator of genetic action of the mutagen. Singh et al. (2013) reported that increased Gy doses provided higher frequency of chlorophyll mutants in cowpea when compared to other mutagens such as EMS. Girija and Dhanavel (2009) outlined the effectiveness and efficiency of mutagens for selection of mutants with economic traits. The authors suggested that for effective phenotypic selection the mutation treatment should not yield unintended damages including chromosomal aberrations, physiological and toxic effects, which reduce cell survival and ultimately eliminate the mutation. Despite its negative effects on the early stages of crop growth, chlorophyll mutants are important in mutation breeding programs. Tulmann Neto et al. (2011) reported that the chlorophyll mutants were used in evaluation of the genetic effects and sensitivity of various mutagens on crops. These results are in agreement with Goyal and Khan (2010) whose studies indicated that the incidence of chlorophyll mutants were higher with increased Gy doses in earlier selection generations.

In the present study, mutants at the M_2_ were genetically diverse owing to phenotypic segregation. The genetic diversity assessed in these mutants were tall/dwarf plant heights, early/late maturity, leaf shapes, branching habit, GH, PS, FC, SC and texture, seed weight and yield (**Tables 4–6**). Both the qualitative and quantitative parameters measured in the study were useful for selection of cowpea mutants. According to Maluszynski et al. (2009), induced genetic polymorphism among initial cells of the sporogenic layer influences the segregation ratio in the M_2_ generation. However, mutations of cells of somatic tissues are not transferred to the next generation. Gnanamurthy et al. (2012) stipulated that easily detectable mutants characteristics are phenotypically visible and morphologically distinct with qualitatively inherited genetic changes. These changes occur due to the effect of few major genes or oligogenes yielding macro mutations. In this study, some macro mutations observed were the changes in flower and SC. Micro mutations are the result of polygenes each with minor genetic effect showing quantitative inheritance. The effect and inheritance of minor genes is detected using quantitative genetic parameters and statistical methods (Singh et al., 2006). In the current study, short plant height and one seed per pod mutants were recorded in all the breeding lines mostly at the M_3_ generation. Single seeded pods were also reported by Girija and Dhanavel (2009).

In the present study, other main phenotypic changes observed were increased NMB especially in mutants with spreading GH. Mutants with bushy GH had reduced number of branches per plant. These characters are indicated to be associated with some physiological properties of the plant including leaf senescence and indeterminate GH (Hall, 2004; Martins et al., 2014). It is reported that characteristics altered through mutation breeding can be combined through the conventional breeding to improve crop performance and drought adaptation (Ehlers and Hall, 1997). The present study found that Nakare mutants had a maximum of 23 main branches per plant, while the comparative control had nine main branches (**Table 3**). According to previous studies (Singh et al., 2003, 2013), the spreading and semi-spreading cowpea types yielded less grain and more fodder when planted in closer spaced rows. The present study found that mutation treatment did not significantly affect the number of days taken to germination, hence all the breeding lines germinated 3 days after planting (**Tables 3–6**). The mutation treatment had positive effect on the number of days taken to 50% flowering whereby some of the breeding lines flowered 11 days before the control. Bira mutants subjected to irradiation of 300 Gy flowered 80 days after planting (**Table 3**). Maluszynski et al. (2009) suggested that a high dose of a mutagen should yield delayed maturity. Dhanavel et al. (2008) repooted that mutagenesis resulted into variation in plant development including the number of days taken to maturity. According to Singh et al. (2003), these variations are important to the farmers and the breeders allowing choices of planting time. The breeder will have a choice from a larger breeding stock for various breeding traits and purposes.

Significant observations made in the present study were increased PL and seed yield measured during the M_3_ to M_5_ in all the breeding lines. Goyal and Khan (2010) reported that mutations caused increased PL in some of the cowpea lines. Pod size may contribute to increased seed yield. The number of grains per pod increases with increased PL though this may be associated with reduced total biomass (Singh et al., 2003). Other major effects of the mutation observed in the present study were the range of variations in SC. A mosaic of SCs were noted including white, brown, chocolate, red, speckled, cream, and black. Dhanavel et al. (2008) reported various SCs due to mutational events. The present findings suggested that the NMB per plant, NPP, number of grains per pod, 100-seed weight and seed yield per plant reduced significantly with increased concentration of irradiation doses. These findings are in agreement to the studies of Girija and Dhanavel (2009), who reported that mutagenesis is associated with negative and positive phenotypic effects for selection.

**FIGURE 6:**
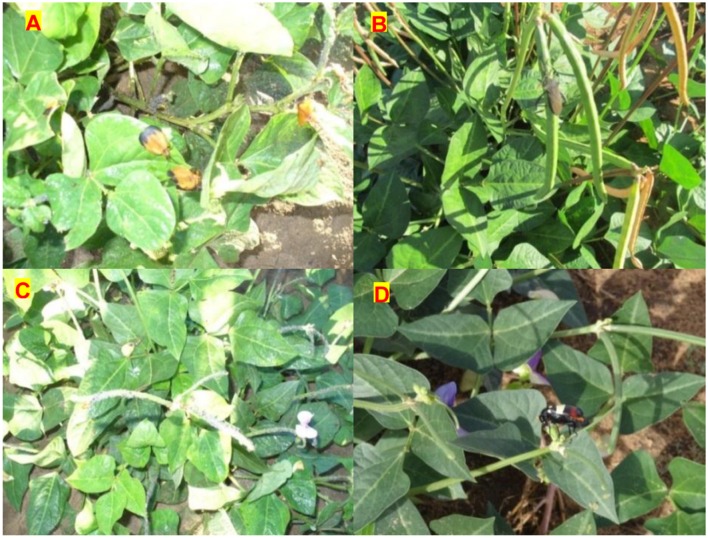
**Common insect pests **(A)** Spiny brown bugs *Clavigralla* sp., **(B)** Coreid bug *Anoplocnemis curvipes*, **(C)** Aphids *Aphis craccivora* Koch and Blister **(D)** Beetle *Mylabris phalerata* observed among the M_5_ mutants at Bagani, and Omahenene Research Stations concurrently**.

The present study demonstrated that most characters of cowpea which are of interest to plant breeders can be altered through mutations using the gamma irradiation technique. Furthermore, new plant attributes were created in the high yielding and well adapted local cowpea varieties. Various pests were observed on mutant cowpea during this study **Figure 6**. Therefore, there is a need to breed for insect pest tolerance in cowpea. Timko et al. (2007) suggested that the future of cowpea improvement programs should focus on breeding for pests and diseases resistance and other desirable traits such as early maturity, photoperiod insensitivity, suitable plant type, seed quality and yield. Overall, the present study made extensive phenotypic selections of mutants from the M_2_ to M_5_ generations and identified promising genotypes. The selected mutants’ are recommended for adaptability and stability tests across representative agro-ecologies for large-scale production or breeding in Namibia or similar environments. The novel cowpea genotypes selected through the study are valuable genetic resources for genetic enhancement and breeding.

## Author Contributions

LH and HS designed the research. The experiments were carried out by LH under the supervision of HS. The manuscript was prepared by LH, HS, and HG.

## Conflict of Interest Statement

The authors declare that the research was conducted in the absence of any commercial or financial relationships that could be construed as a potential conflict of interest.
